# Sex-specific association between fibroblast growth factor 21 and type 2 diabetes: a nested case-control study in Singapore Chinese men and women

**DOI:** 10.1186/s12986-017-0216-0

**Published:** 2017-09-30

**Authors:** Yeli Wang, Woon-Puay Koh, Jian-Min Yuan, An Pan

**Affiliations:** 1 Saw Swee Hock School of Public Health, National University of Singapore and National University Health System, Singapore, 117549 Singapore; 2 Duke-NUS Medical School, Singapore, 169857 Singapore; 3 UPMC Hillman Cancer Center, University of Pittsburgh, Pittsburgh, PA 15232 USA; 4 Department of Epidemiology, Graduate School of Public Health, University of Pittsburgh, Pittsburgh, PA 15261 USA; 5 Department of Epidemiology and Biostatistics, Ministry of Education Key Laboratory of Environment and Health and State Key Laboratory of Environmental Health (incubation), School of Public Health, Tongji Medical College, Huazhong University of Science and Technology, Wuhan, Hubei Province 430030 China

**Keywords:** Fibroblast growth factor 21, Nested case-control study, Type 2 diabetes, Prospective study

## Abstract

**Background:**

Fibroblast growth factor 21 (FGF-21) is mainly secreted by liver and has been reported to be involved in the pathogenesis of type 2 diabetes. Some prospective studies have shown a positive association between FGF-21 and diabetes risk. However, no study has examined whether the association differed by sex, which has been reported between FGF-21 and atherosclerosis. Therefore, we prospectively evaluated the sex-specific association between FGF-21 and diabetes in a Chinese population.

**Methods:**

Serum FGF-21 concentration was measured in a case-control study comprising of 251 incident diabetes cases and 251 age-sex-matched controls nested within a prospective population-based cohort, the Singapore Chinese Health Study. At blood collection between 1999 and 2004, participants were free of diagnosed diabetes, cardiovascular disease, and cancer. Incident self-reported diabetes cases were identified at follow-up II interview (2006–2010). Odds ratio (OR) and 95% confidence interval (CI) were calculated using multivariable logistic regression models.

**Results:**

After adjustment for risk biomarkers of diabetes including lipids, liver enzymes and inflammatory marker, the OR of type 2 diabetes with per one unit increment in log FGF-21 concentration was 1.16 (95% CI 0.90–1.50). Significant interaction was found with sex (*P*-interaction = 0.029): the OR (95% CI) was 1.50 (1.00-2.25) in women and 0.89 (0.52–1.53) in men.

**Conclusions:**

Higher serum FGF-21 level was associated with an increased risk of diabetes in Chinese women but not in men. The sex difference in the association between FGF-21 and diabetes risk deserves further investigation and replication in other populations.

**Electronic supplementary material:**

The online version of this article (10.1186/s12986-017-0216-0) contains supplementary material, which is available to authorized users.

## Background

Fibroblast growth factor 21 (FGF-21) is a hormone secreted mainly by the liver, as well as by adipose tissue, pancreas, and skeletal muscle [1]. FGF-21 has increasingly attracted attention recently due to its potential beneficial role in glucose homeostasis and lipid metabolism [2]. Animal studies have shown that FGF-21 ameliorates hyperglycemia, hyperlipidemia, and insulin resistance [3], and may thus prevent the development of type 2 diabetes.

Despite the favorable metabolic changes observed in animal studies, cross-sectional studies in humans have found that circulating FGF-21 levels were paradoxically elevated with conditions of metabolic dysregulations such as metabolic syndrome [4] and type 2 diabetes [5], although a temporal relationship cannot be determined from these studies. So far, only three prospective cohort studies with relatively small cases numbers (patients with diabetes ranged from 54 to 123) have been conducted, and all reported a positive association between higher FGF-21 levels and increased risk of type 2 diabetes [6–8]. However, it is largely unknown whether nonalcoholic fatty liver disease (NAFLD), a condition associated with both diabetes and FGF-21 levels [9], may explain or modify the association between FGF-21 and diabetes risk. Moreover, recent studies have reported an interaction between FGF-21 and sex in its association with atherosclerosis and bone mineral density [10–12], with an association observed in women but not in men, but this has not been studied in the context of diabetes. Furthermore, two studies have examined whether FGF-21 added substantial value in predicting diabetes risk, and the results were inconsistent [6, 8]. While one study reported that the predictive utility of FGF-21 was as good as the oral glucose tolerance test (OGTT) [8], the other study found that including FGF-21 did not improve diabetes prediction [6].

In this nested case-control study within the Singapore Chinese Health Study, we studied the association between FGF-21 and risk of type 2 diabetes with adjustment of established diabetes risk factors including two liver enzymes alanine transaminase (ALT) and gamma-glutamyltransferase (GGT). ALT and GGT could serve as surrogate markers of NAFLD [13], and we have previously reported a positive association between the two liver enzymes with diabetes risk [14]. We also performed stratified analysis to explore the potential heterogeneity among different subgroups, particularly sex. Additionally, we assessed the incremental value of FGF-21 in diabetes risk prediction over established risk factors in this population.

## Methods

### Study population

The design of the Singapore Chinese Health Study has been described in detail previously [15]. Briefly, the prospective cohort was established between 1993 and 1998, and recruited 63,257 Chinese adults aged between 45 and 74 years. Baseline information on diet, lifestyle habits and medical history was collected at recruitment by an in-person interview. Between 1999 and 2004, follow-up I interviews were conducted via telephone. Among 52,322 participants who were re-contacted successfully, a total of 32,535 individuals donated their bio-specimens. Between 2006 and 2010, follow-up II interviews were conducted via telephone, and a total of 39,528 participants were re-contacted successfully. The study protocol was approved by the Institutional Review Boards at the National University of Singapore and the University of Pittsburgh. Informed consent was provided and completed by each participant at the baseline interview.

### Ascertainment of diabetes and other covariates

At baseline and both follow-up interviews, history of physician-diagnosed diabetes was asked by the question: “Have you been told by a doctor that you have diabetes?” If the participant answered “yes”, he or she was also asked for the age of first diagnosis. The robustness and accuracy of the self-reported diabetes data has been confirmed in a validation study [16].

Height and body weight were self-reported at baseline and both follow-ups. Body mass index (BMI) was calculated as weight (kilograms) divided by height (meters) squared. For those participants with missing height or weight, BMI was calculated using imputed data derived from the linear regression equation: Weight = y-intercept + gradient × height. The values for the y-intercept and gradient were derived from gender-specific weight-height regression lines, which were obtained from all cohort participants with reported heights and weights.

### Establishment of nested case-control study

For the current analysis, we established a nested case-control study of 251 incident cases and 251 matched controls within this cohort. All cases and controls were free of physician-diagnosed diabetes, cardiovascular disease and cancer at baseline interview as well as the time of blood collection during 1999 and 2004. Among 571 participants who subsequently reported to be diagnosed with diabetes during follow-up II visit (2006–2010), we selected 292 cases who had hemoglobin A1c (HbA_1c_) levels <6.5% at blood donation to exclude undiagnosed diabetes. Controls were chosen from the remaining participants who did not develop diabetes or cardiovascular disease at follow-up II, and were matched for age (±3 years), date of blood collection (±6 months), sex (men, women), and dialect group (Cantonese, Hokkien) with the cases on a 1:1 ratio. Furthermore, controls were considered eligible if their baseline HbA_1c_ levels were less than 6.0%. Some participants with insufficient serum samples (*n* = 37) or extreme FGF-21 levels (>3 standard deviation [SD], *n* = 4) were excluded, leaving a total of 251 case-control pairs for the present study. The flowchart of the study design is shown in Additional file 1: Figure S1.

### Laboratory procedures

Twenty-mL random morning blood was collected from each consenting participant and separated into plasma, serum, red blood cells, and buffy coat, and stored in −80 °C freezers. Serum concentrations of FGF-21 and plasma levels of adiponectin were measured by ELISA/Evolis (Bio-Rad Laboratories, Hercules, CA). Plasma concentrations of high-sensitivity C-reactive protein (hs-CRP), total cholesterol (TC), triglycerides (TG), high-density lipoprotein cholesterol (HDL-C), ALT and serum levels of GGT were measured via colorimetric method on a chemistry analyzer (AU5800 Analyzer, Beckman Coulter, Brea, CA). HbA_1c_ levels in red blood cells were measured by HPLC method using Bio-Rad Variant II™ System (Bio-Rad Laboratories, Hercules, CA).

### Statistical analysis

Because of the different distributions between men and women, baseline characteristics were presented for men and women separately. For prospective analyses, study participants were divided into quartiles according to the sex-specific distribution of FGF-21 levels among control participants, and the lowest quartile served as the reference group. Conditional logistic regression models were used to calculate the odds ratio (OR) and corresponding 95% confidence interval (CI) between FGF-21 and diabetes. Model 1 was adjusted for age (continuous), smoking (never, ever smoker), alcohol intake (never, ever drinker), weekly moderate-to-vigorous  physical activity (<0.5, ≥0.5 h/week), education level (primary school and below, secondary or above), history of hypertension (yes, no), fasting status (yes, no) and BMI (continuous). In addition, since FGF-21 levels were associated with hs-CRP levels [17], dyslipidemia [3], and nonalcoholic fatty liver disease [17], we further adjusted for the metabolic biomarkers (hs-CRP, TG, HDL-C, GGT, and ALT) both in quartiles and as continuous variables to examine their impact on the association between FGF-21 and diabetes. Because of the significant interaction with sex, we repeated the abovementioned analysis in men and women separately, and we have additionally adjusted for menopausal status in women. We then used restricted cubic spline regression with 4 knots at 5%, 35%, 65% and 95% percentiles of original value of FGF-21 to examine a possible non-linear relation between FGF-21 and diabetes risk. When the nonlinear hypothesis was rejected, we also calculated the diabetes risk associated with per 1 unit increment in log FGF-21 levels, in order to compare our results with previous studies [6, 7]. Moreover, we tested potential interactions with age (<60 or ≥60 years), sex (men, women), fasting status (yes, no), BMI (<23 or ≥23 kg/m^2^), physical activity (<0.5 or ≥0.5 h/week), plasma levels of hs-CRP, GGT, ALT, TG, or HDL-C (above or below median levels of each biomarker) by adding an interaction term (each binary variable × log-transformed FGF-21) to the regression models in the men and women separately. Potential interaction with menopausal status was additionally tested in women. In the stratified analysis by non-matching variables, unconditional logistic regression models were used with additional adjustment for sex and dialect group (Cantonese, Hokkien).

The predictive utility of FGF-21 for diabetes prediction was subsequently examined. The optimal cutoff value was derived by using receiver-operating characteristic (ROC) analysis and Youden index [18]. Base model 1 included age and BMI; base model 2 additionally included smoking status, history of hypertension, and levels of TG, HDL-C, and random glucose. In addition, we built the base model 3 to further include adiponectin and hs-CRP. The improvement in discrimination between the parsimonious model and the model plus binary FGF-21 was examined by comparing area under receiver-operating characteristic curve (AUC) using DeLong’s method [19]. Moreover, we used the category-free net reclassification improvement (NRI) and integrated discrimination improvement (IDI) statistics recommended by Pencina et al. [20, 21]. Furthermore, we used Akaike information criteria (AIC) to assess the goodness-of-fit of all models, where lower AICs indicate better model fit. Analyses were performed with Stata software, version 14.0 (Stata Corp, College Station, Texas). Two-sided *P* values <0.05 were considered to be statistically significant.

## Results

Among the cases, the mean age of diagnosis of incident type 2 diabetes (SD) was 63.2 (6.4) years and the mean duration (SD) between blood donation and diagnosis was 4.0 (1.7) years. The sex-specific baseline characteristics of cases and controls stratified by sex are shown in Table 1. In both men and women, cases had higher BMI and were more likely to have hypertension compared to matched controls. No significant differences were found for education levels, smoking status and alcohol consumption. In addition, 54 women (20%) were premenopausal and 214 women (80%) were at postmenopausal status. For blood biomarkers in both men and women, cases had higher levels of FGF-21, HbA_1c_, hs-CRP, TG, GGT, ALT, but lower levels of adiponectin and HDL-C. Among both cases and controls, serum FGF-21 levels were positively correlated with ALT, GGT, TG and hs-CRP, and negatively correlated with HDL-C and adiponectin (Additional file 1: Table S1). Similar pattern was found in men and women (data not shown).

**Table 1:** Baseline characteristics and liver enzymes of diabetes cases and matched controls in men and women, the Singapore Chinese Health Study^a^

	Men			Women		
	Cases (n = 117)	Controls (*n* = 117)	*P*-value^b^	Cases (*n* = 134)	Controls (n = 134)	*P*-value^b^
Age (years) at blood taken	60.1 ± 6.04	60.2 ± 6.29	–	57.9 ± 5.48	58.4 ± 5.72	–
Dialect (%)			–			–
Cantonese	52 (44.4)	52 (44.4)		74 (55.2)	74 (55.2)	
Hokkien	65 (55.6)	65 (55.6)		60 (44.8)	60 (44.8)	
Body mass index (kg/m^2^)	24.9 ± 3.58	22.9 ± 3.43	<0.001	24.7 ± 3.79	22.4 ± 3.42	<0.001
Postmenopausal status	–	–	–	106 (79.1)	108 (80.6)	0.76
Level of education (%)			0.58			0.82
No formal education	6 (5.13)	7 (5.98)		24 (17.9)	26 (19.4)	
Primary school	58 (49.6)	50 (42.7)		57 (42.5)	60 (44.8)	
Secondary and above	53 (45.3)	60 (51.3)		53 (39.6)	48 (35.8)	
History of Hypertension (%)	61 (52.1)	29 (24.8)	<0.001	63 (47.0)	28 (20.9)	<0.001
Cigarette smoking (%)			0.28			0.15
Never smokers	54 (46.2)	47 (40.2)		124 (92.5)	130 (97.0)	
Former smoker	27 (23.1)	38 (32.5)		6 (4.5)	1 (0.8)	
Current smokers	36 (30.8)	32 (27.4)		4 (3.0)	3 (2.2)	
Weekly moderate-to-vigorous activity (%)			0.20			0.02
< 0.5 h/week	89 (76.1)	90 (76.9)		107 (79.8)	104 (77.6)	
0.5–3.9 h/week	21 (18.0)	14 (12.0)		23 (17.2)	15 (11.2)	
≥ 4 h/week	7 (5.98)	13 (11.1)		4 (3.0)	15 (11.2)	
Alcohol Intake (%)			0.98			0.35
Abstainers	96 (82.1)	95 (81.2)		127 (94.8)	126 (94.0)	
Weekly drinkers	17 (14.5)	18 (15.4)		7 (5.2)	6 (4.5)	
Daily drinkers	4 (3.42)	4 (3.42)		0	2 (1.5)	
Fasting status (yes)	34 (29.1)	32 (27.4)	0.77	40 (29.9)	35 (26.1)	0.50
FGF-21, pg/mL	212 (133–350)	179 (82–312)	0.056	222 (115–365)	146 (65–259)	<0.001
GGT, IU/L	34 (26–49)	27 (20–40)	<0.001	25 (18–41)	19 (14–28)	<0.001
ALT, IU/L	29 (21–39)	23 (17–29)	<0.001	23 (17–32)	18 (14–23)	<0.001
TC, mmol/L	5.03 ± 0.91	5.02 ± 0.69	0.92	5.32 ± 0.80	5.30 ± 0.91	0.80
HDL-C, mmol/L	0.96 ± 0.19	1.09 ± 0.25	<0.001	1.18 ± 0.26	1.35 ± 0.30	<0.001
LDL-C, mmol/L	3.04 ± 0.76	3.09 ± 0.68	0.59	3.18 ± 0.75	3.27 ± 0.78	0.32
TG, mmol/L	2.1 (1.5–2.9)	1.6 (1.2–2.3)	<0.001	2.1 (1.4–2.7)	1.4 (0.9–1.9)	<0.001
Adiponectin, μg/mL	6.53 ± 2.64	7.86 ± 2.78	<0.001	7.93 ± 3.19	10.3 ± 3.80	<0.001
Hs-CRP, mg/L	1.6 (0.8–2.9)	1.2 (0.6–1.9)	0.02	1.8 (1.0–3.5)	1.2 (0.7–2.1)	<0.001
Random insulin, mIU/L	15.4 (7.0–37.3)	8.0 (4.4–20.5)	<0.001	14.1 (8.1–35.4)	7.7 (4.2–23.5)	<0.001
Random glucose, mmol/L	5.1 (4.4–6.6)	4.5 (4.0–5.1)	<0.001	5.3 (4.4–6.1)	4.5 (3.9–5.3)	<0.001
HbA_1c_, %	5.9 (5.6–6.2)	5.6 (5.4–5.7)	<0.001	5.9 (5.7–6.2)	5.6 (5.4–5.7)	<0.001
HbA_1c_, mmol/mol	41 (38–44)	38 (36–39)	<0.001	41 (39–44)	38 (36–39)	<0.001

^a^Data are expressed as mean ± standard deviation for continuous variables (normally distributed) and median (interquartile range) for continuous variables (skewed distributed), and n (percentage) for categorical variables. Cases and controls are matched on age at blood taken (±3 years), gender, dialect, and date of blood collection (±6 months)

^b^ *P* values based on the chi-square test for categorical variables, student’s t-test and Mann-Whitney test for continuous variable

**Fig. 1:**
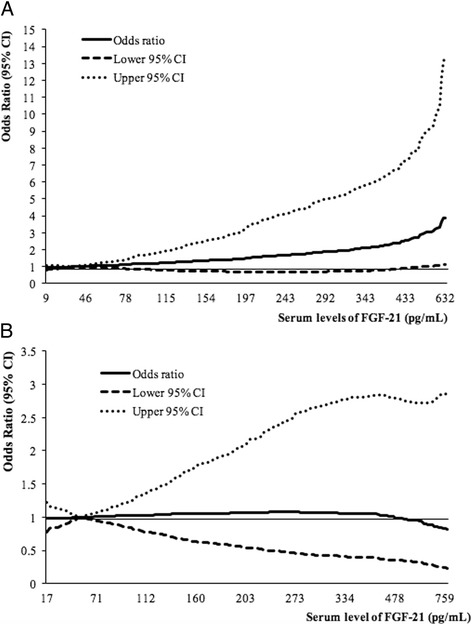
Spline analysis of the association between serum levels of FGF-21 and incident type 2 diabetes in women (**a**) and men (**b**). The *Solid line* represents point estimates of relative risk for the association between FGF-21 and incident type 2 diabetes, and the *dotted lines* represent the upper and lower bound of 95% CI. Study participants with the lowest and highest 1% of FGF-21 were excluded to minimize the potential impact of outliers. Cubic spline analysis was used to examine the association between FGF-21 concentrations and risk of developing type 2 diabetes using the most fully-adjusted models from reported studies. *P* for nonlinearity =0.09 in women (**a**) *P* for nonlinearity =0.86 in men (**b**) in the cubic spline regression model

The association between FGF-21 and risk of type 2 diabetes is presented in Table 2. In the total study population, higher levels of serum FGF-21 were significantly associated with increased diabetes risk after adjustment for age, sex, lifestyle factors, fasting status and BMI; the OR (95% CI) comparing the highest versus lowest quartile was 2.70 (1.33–5.50; *P*-trend = 0.015). However, after mutual adjustment for quartiles of hs-CRP, TG, HDL-C, GGT, and ALT, the association became statistically non-significant in model 2 (OR 1.75; 95% CI 0.76–4.01; *P*-trend = 0.23). Significant interaction was found with sex (*P*-interaction = 0.029), and the association was evident in women only (OR 4.19; 95% CI 1.07–16.5; *P*-trend = 0.036) but not in men (OR 1.20; 95% CI 0.24–5.94; *P*-trend = 0.70) comparing the extreme quartiles of FGF-21 levels in model 2. In addition, cubic spline regression model suggested a linear relationship between FGF-21 and T2D risk in both men (*P* for nonlinearity =0.86) and women (*P* for nonlinearity =0.09) (Fig. 1). Given the linear association, we further estimated for the every 1-log pg/mL increment in FGF-21 levels, the ORs (95% CIs) for diabetes in model 2 were 1.16 (0.90–1.50) in the total study samples, 0.89 (0.52–1.53) in men and 1.53 (1.02–2.29) in women. In a sensitivity analysis, we used continuous values of blood biomarkers instead of quartiles in the multivariable adjustment, and the results were materially changed: the ORs (95% CIs) for diabetes with per-log increment in FGF-21 levels were 1.15 (0.91–1.45) in the total population, 0.80 (0.53–1.23) in men and 1.43 (1.00–2.05) in women (data not shown). In addition, further adjustment for menopausal status in women had little impact on the association between FGF-21 and diabetes (OR per log FGF-21: 1.50; 95% CI 1.00–2.25). Moreover, no statistically significant interactions were found with other variables in the total study sample (Table 3), men and women (Table 4); however, the sample size in the stratified analyses was much smaller.

**Table 2:** Odds ratios (95% confidence intervals) of type 2 diabetes associated with different levels of FGF-21 in men and women, the Singapore Chinese Health Study

Variables	Quartiles of FGF-21				*P* for trend^a^	Per 1 log increment
	Q1	Q2	Q3	Q4		
Whole dataset						
Median (range)	47 (7–75)	116 (76–157)	212 (158–288)	411 (289–1607)		
Cases/controls	31/64	61/63	64/62	95/62		
Model 1^b^	1.00	1.96 (1.00–3.85)	1.77 (0.94–3.33)	2.70 (1.33–5.50)	0.015	1.28 (1.03–1.59)
Model 1^b^ + hs-CRP	1.00	1.73 (0.87–3.44)	1.69 (0.89–3.21)	2.56 (1.24–5.31)	0.018	1.27 (1.01–1.58)
Model 1^b^ + TG, HDL-C	1.00	1.76 (0.85–3.67)	1.47 (0.74–2.94)	1.91 (0.87–4.20)	0.20	1.19 (0.93–1.52)
Model 1^b^ + ALT, GGT	1.00	1.79 (0.88–3.63)	1.77 (0.90–3.49)	2.10 (0.98–4.50)	0.09	1.20 (0.95–1.51)
Model 2^c^	1.00	1.53 (0.70–3.32)	1.49 (0.72–3.10)	1.75 (0.76–4.01)	0.23	1.16 (0.90–1.50)
Men						
Median (range)	59 (7–82)	133 (83–179)	240 (180–312)	483 (317–1460)		
Cases/controls	16/30	32/29	32/29	37/29		
Model 1^b^	1.00	2.00 (0.76–5.28)	1.43 (0.55–3.70)	1.66 (0.53–5.21)	0.68	0.95 (0.65–1.38)
Model 1^b^ + hs-CRP	1.00	1.80 (0.65–4.97)	1.35 (0.51–3.55)	1.71 (0.52–5.65)	0.63	0.95 (0.64–1.41)
Model 1^b^ + TG, HDL-C	1.00	1.44 (0.50–4.17)	1.33 (0.47–3.83)	1.21 (0.33–4.39)	0.88	0.94 (0.60–1.46)
Model 1^b^ + ALT, GGT	1.00	1.66 (0.56–4.98)	1.59 (0.54–4.69)	1.27 (0.33–4.80)	0.81	0.85 (0.55–1.31)
Model 2^c^	1.00	1.32 (0.36–4.80)	1.67 (0.47–5.95)	1.20 (0.24–5.94)	0.70	0.89 (0.52–1.53)^e^
Women						
Median (range)	30 (7–65)	107 (65–146)	197 (147–259)	375 (260–1607)		
Cases/controls	15/34	29/34	32/33	58/33		
Model 1^b^	1.00	2.13 (0.76–5.97)	2.15 (0.87–5.28)	4.73 (1.69–13.2)	0.004	1.60 (1.16–2.19)
Model 1^b^ + hs-CRP	1.00	1.74 (0.60–5.03)	2.11 (0.84–5.33)	4.07 (1.42–11.7)	0.006	1.56 (1.13–2.15)
Model 1^b^ + TG, HDL-C	1.00	2.28 (0.67–7.74)	1.87 (0.67–5.20)	4.37 (1.31–14.6)	0.033	1.49 (1.02–2.17)
Model 1^b^ + ALT, GGT	1.00	2.25 (0.72–7.03)	2.28 (0.86–6.05)	4.30 (1.38–13.4)	0.014	1.54 (1.10–2.16)
Model 2^c^	1.00	1.90 (0.50–7.26)	2.28 (0.73–7.14)	4.19 (1.07–16.5)	0.036	1.53 (1.02–2.29)^e^
Model 3^d^	1.00	1.81 (0.46–7.14)	2.27 (0.72–7.14)	3.88 (0.99–15.3)	0.047	1.50 (1.00–2.25)

^a^Linear trend was tested by using the median level of each quartile of FGF-21

^b^Model 1: adjusted for age at blood taken (continuous), smoking (never, ever smoker), alcohol intake (never, ever drinker), weekly moderate-to-vigorous activity (<0.5, ≥0.5 hours/week), education level (primary school and below, secondary or above), history of hypertension (yes, no), fasting status (yes, no), and body mass index (continuous)

^c^Model 2: Model 1 plus hs–CRP (mg/L), TG (mmol/L), HDL-C (mmol/L), GGT (IU/L) and ALT (IU/L) (all in quartiles)

^d^Mode 3: Model 2 plus menopausal status (premenopausal, postmenopausal status)

^e^The *P*-interaction =0.029 between FGF-21 and sex associated with incident type 2 diabetes risk

**Table 3:** Odds ratios (95% confidence intervals) of incident type 2 diabetes by stratified analysis in the total samples, the Singapore Chinese Health Study^a^

Variables	Per 1 log increment of FGF-21		*P*-interaction
	Cases/ controls	OR (95% CI)	
Age (year)			
< 60	151/150	1.35 (1.04–1.76)	0.39
≥ 60	100/101	1.20 (0.86–1.67)	
BMI, kg/m^2^			
< 23	117/117	1.31 (0.99–1.73)	0.96
≥ 23	134/134	1.26 (0.94–1.70)	
Physical activity			
< 0.5 h/week	84/148	1.42 (1.12–1.80)	0.46
≥ 0.5 h/week	167/103	1.00 (0.65–1.54)	
Fasting status			
Fasting	196/194	1.25 (0.99–1.57)	0.64
Non-fasting	55/57	1.39 (0.87–2.21)	
Hs-CRP, mg/L			
< 1.4	74/67	1.38 (1.03–1.85)	0.93
≥ 1.4	177/184	1.34 (1.01–1.78)	
GGT, IU/L			
< 30	102/140	1.21 (0.91–1.61)	0.18
≥ 30	149/111	1.37 (1.00–1.89)	
ALT, IU/L			
< 26	96/147	1.26 (0.93–1.69)	0.51
≥ 26	155/104	1.30 (0.96–1.77)	
TG, mmol/L			
< 1.815	91/140	1.08 (0.82–1.42)	0.18
≥ 1.815	160/111	1.42 (1.02–1.97)	
HDL-C, mmol/L			
< 0.99	94/157	1.12 (0.80–1.56)	0.87
≥ 0.99	157/94	1.26 (0.96–1.65)	

^a^Odds ratios were estimated after adjusting for adjusted for age at blood taken (continuous), smoking (never, ever smoker), alcohol intake (never, ever drinker), weekly moderate-to-vigorous activity (<0.5, ≥0.5 hours/week), education level (primary school and below, secondary or above), history of hypertension (yes, no), fasting status (yes, no) and BMI (continuous), except for stratifying factors

**Table 4:** Odds ratios (95% confidence intervals) of type 2 diabetes by stratified analysis in men and women separately, the Singapore Chinese Health Study^a^

Variables	Per 1 log increment of FGF-21		*P*-interaction
	Cases/ controls	OR (95% CI)	
Men			
Age (year)^a^			
< 60	65/64	1.17 (0.74–1.86)	0.43
≥ 60	52/53	0.88 (0.53–1.44)	
BMI^a^, kg/m^2^			
< 23	33/67	0.94 (0.58–1.51)	0.65
≥ 23	84/50	1.12 (0.69–1.81)	
Physical activity^a^			
< 0.5 h/week	89/90	1.19 (0.81–1.74)	0.46
≥ 0.5 h/week	28/27	0.61 (0.26–1.44)	
Fasting status^a^			
Fasting	83/85	0.98 (0.66–1.44)	0.91
Non-fasting	34/32	1.26 (0.62–2.56)	
Hs-CRP^a^, mg/L			
< 1.4	51/64	1.08 (0.66–1.75)	0.79
≥ 1.4	66/53	1.07 (0.65–1.76)	
GGT^a^, IU/L			
< 30	47/68	0.81 (0.42–1.54)	0.21
≥ 30	70/49	1.11 (0.73–1.67)	
ALT^a^, IU/L			
< 26	46/70	0.83 (0.47–1.48)	0.58
≥ 26	71/47	1.09 (0.69–1.70)	
TG^a^, mmol/L			
< 1.815	49/68	0.65 (0.39–1.08)	0.61
≥ 1.815	68/49	1.56 (0.88–2.77)	
HDL-C^a^, mmol/L			
< 0.99	75/41	1.22 (0.79–1.88)	0.95
≥ 0.99	42/76	0.82 (0.41–1.61)	
Women			
Age (year)^b^			
< 60	86/86	1.48 (1.06–2.07)	0.74
≥ 60	48/48	1.44 (0.86–2.41)	
BMI^b^, kg/m^2^			
< 23	51/81	1.53 (1.06–2.22)	0.62
≥ 23	83/53	1.43 (0.95–2.14)	
Physical activity^b^			
< 0.5 h/week	107/104	1.62 (1.18–2.22)	0.34
≥ 0.5 h/week	27/30	1.29 (0.71–2.37)	
Fasting status^b^			
Fasting	94/99	1.43 (1.06–1.92)	0.56
Non-fasting	40/35	1.74 (0.86–3.53)	
Menopausal status^b^			
Premenopausal	26/28	1.14 (0.61–2.12)	0.50
Postmenopausal	108/106	1.57 (1.15–2.14)	
Hs-CRP^b^, mg/L			
< 1.4	51/76	1.49 (0.99–2.24)	0.67
≥ 1.4	83/58	1.33 (0.88–2.01)	
GGT^b^, IU/L			
< 30	49/78	1.34 (0.96–1.86)	0.10
≥ 30	85/56	1.98 (1.08–3.63)	
ALT^b^, IU/L			
< 20	45/80	1.36 (0.95–1.95)	0.25
≥ 20	89/54	2.05 (1.20–3.49)	
TG^b^, mmol/L			
< 1.66	48/86	1.42 (0.97–2.07)	0.52
≥ 1.66	86/48	1.57 (0.99–2.50)	
HDL-C^b^, mmol/L			
< 1.23	83/50	1.16 (0.59–2.32)	0.69
≥ 1.23	51/84	1.40 (1.02–1.91)	

^a^Odds ratios were estimated after adjusting for adjusted for age at blood taken (continuous), smoking (never, ever smoker), alcohol intake (never, ever drinker), weekly moderate-to-vigorous activity (<0.5, ≥0.5 hours/week), education level (primary school and below, secondary or above), history of hypertension (yes, no), fasting status (yes, no) and BMI (continuous), except for stratifying factors

^b^Odds ratio were adjusted for the abovementioned variables plus menopausal status

Since we only found a significant association among women, we limited the analysis for the predictive utility of FGF-21 to women only. The best cutoff predictive value for risk of diabetes was 123 pg/mL using Youden index in the ROC analysis. The sensitivity and specificity of the cutoff point were 75.7% and 41.4%, respectively. The predictive performance of FGF-21 is presented in Table 5 and Additional file 1: Table S2. In all base models, addition of binary FGF-21 did not significantly improve the AUC (AUC changes range from 0.004 to 0.018, *P* values range from 0.11 to 0.56). However, adding binary FGF-21 resulted in statistically significant NRI (NRIs range from 0.358 to 0.388, all *P* < 0.01) and IDI (IDIs range from 0.013 to 0.028, *P* values range from 0.009 to 0.052) in all 3 models.

**Table 5:** Summary statistics to assess binary FGF-21 in predicting incident type 2 diabetes risk among female participants, the Singapore Chinese Health Study^a^

Variable	Multivariable models^b^			
	Discrimination (AUC [95% CI])	Calibration (AIC)	NRI	IDI
Base model 1^c^	0.622 (0.555–0.689)	156		
Base model 1^c^ + FGF-21	0.640 (0.574–0.706)^f^	149	0.358 (0.118–0.598)	0.028 (0.007–0.048)
Base model 2^d^	0.764 (0.707–0.820)	130		
Base model 2^d^ + FGF-21	0.768 (0.712–0.824)^f^	128	0.358 (0.118–0.598)	0.013 (0.001–0.025)
Base model 3^e^	0.792 (0.739–0.845)	135		
Base model 3^e^ + FGF-21	0.798 (0.746–0.850)^f^	133	0.388 (0.149–0.628)	0.015 (0.001–0.029)

^a^Binary FGF-21 was created using a cutoff point of 123, with a sensitivity of 75.7%, and specificity of 41.4%

^b^Multivariable model adjusted for all the variables included in the base model plus binary FGF-21 (<123 vs. ≥ 123 pg/mL)

^c^Base model 1 included age (continuous) and BMI (continuous)

^d^Base model 2 included variables in base model 1 plus smoking status (never, ever smoker), history of hypertension (yes, no), and levels of TG (mmol/L), HDL-C (mmol/L), and random glucose (mmol/L) (all in quartiles)

^e^Base model 3 included variables in base model 2 plus adiponectin (μg/mL) and hs-CRP (mg/L) (both in quartiles)

^f^Compared to the base model, the *P*-values for the differences of AUC after including FGF-21 to the base model were 0.11 for base model 1, 0.56 for base model 2 and 0.41 for model 3

## Discussion

In this Chinese population in Singapore, we found a strong dose-dependent association between higher serum FGF-21 levels and increased risk of incident type 2 diabetes in women but not in men, and the association was independent of liver enzymes and other diabetes risk factors. In addition, FGF-21 improved diabetes risk reclassification among women.

The positive association between FGF-21 and incident type 2 diabetes found in the current study is in accordance with previous studies [6–8]. A 5.4-year prospective cohort study among 1292 Chinese (73 diabetes cases) in Hong Kong reported an OR of 1.79 (95% CI 1.22–2.64) for the risk of diabetes with per 1 unit increment in log FGF-21 levels, after adjusting for fasting glucose, insulin, TG, HDL-C and hs-CRP [7]. Later, an updated study in the same cohort focusing on diabetes prediction model reported that higher FGF-21 (≥178.2 versus <178.2 pg/mL) was associated with an increased diabetes risk independent of other blood biomarkers (OR, 1.60; 95% CI 1.18–2.16) [8]. So far, only one prospective study (440 Germans with 54 diabetes cases) has examined the impact of liver enzymes on the association between FGF-21 and diabetes [6]. The study found a positive association after adjusting for liver enzymes, although the risk estimate was not reported [6]. In the current study, we adjusted for liver enzymes individually and in combination with other blood biomarkers, and the results remained significant. We also stratified the analysis by liver enzymes and did not find significant effect modification.

A number of experimental evidence has shown that FGF-21 may involve in key etiological pathways leading to diabetes development such as regulation of lipid homeostasis [22], inflammation [23], and development of NAFLD [9]. Increased levels of circulating free fatty acids induced liver FGF-21 secretion by a PPAR-α-dependent pathway [24, 25], and raised FGF-21 lowered TG levels by speeding up lipoprotein catabolism in adipose tissues [26] and by regulating the expression of key genes involved in lipid metabolism [22]. In addition, inflammation in adipose tissue could suppress FGF-21 receptor β-klotho [27], which subsequently attenuated FGF-21 signaling and caused FGF-21 resistance in rodents [23]. However, the current analysis along with prior studies have shown that the association between FGF-21 and diabetes was independent of lipids (TG, HDL-C), inflammatory marker (hs-CRP) and liver enzymes (GGT, ALT) [6–8]. In addition, FGF-21 could reduce glucose levels by inducing expression and secretion of adiponectin [28], and increasing glucose uptake in brown adipose tissues [22] and skeletal muscle [29]. In accordance with the mechanism, we observed a positive correlation between FGF-21 and adiponectin in the current analysis. However, since adiponectin is a consequence rather than a determinant of FGF-21, we did not include adiponectin in the multivariable model. Despite of FGF-21 being a risk factor for diabetes, recent animal study and clinical trials in humans have shown beneficial effects of exogenous FGF-21 on lipid profile, levels of adiponectin, fasting insulin and glucose in obese patients with diabetes [30–32], suggesting FGF-21 may also serve as a potential therapeutic agent for treating type 2 diabetes in humans.

In addition, we have observed sex-interaction in the association between FGF-21 and diabetes in this Chinese population. To the best of our knowledge, no prospective studies have examined the sex interaction with FGF-21 in relation to diabetes. However, three cross-sectional studies have reported significant interactions between FGF-21 and sex in the association with carotid atherosclerosis [10], lower extremity atherosclerotic disease [12] and bone mineral density [11]; in all three studies, significant positive correlations were only observed in women but not in men. The underlying mechanism for the observed sex heterogeneity may be due to different body fat distribution and sex hormone. Compared to men, women have greater accumulation of subcutaneous fat, and higher amount and activity of brown adipose tissue, which was hypothesized to impact whole-body energy metabolism, insulin resistance, and obesity-related diabetes [33]. In addition, animal studies have shown that estrogen increases hepatic production of FGF-21 [34] and enhance the activity of FGF-21 in brown adipose tissue [35]. However, majority of the women were at postmenopausal status in the current study, and no significant interaction was observed with menopausal status. Therefore, whether estrogen played an important role in the association between FGF-21 and diabetes risk remains to be explored.

A prospective study in Hong Kong reported that addition of FGF-21 to a diabetes prediction model comprising of age, family history of type 2 diabetes, smoking, hypertension, BMI, dyslipidemia and fasting glucose showed statistically significant improvement in AUC from 0.797 to 0.819, and its performance in diabetes prediction is comparable to the OGTT [8]. In contrast, another prospective cohort study in a German population reported that addition of FGF-21 to a base model including age, sex, BMI and time of follow-up did not yield statistically significant increment in AUC [6]. In the current study, we did not observe significant improvement in AUC change after adding FGF-21 into the models. Since AUC has the limitation of being relatively insensitive to model improvement [36], we further applied other statistical methods such as NRI and IDI that have shown to be useful in assessing the predictive utility of novel biomarkers [20, 21]. As a result, including FGF-21 in the model significantly improved NRI and IDI, and the best cut-off value (123 pg/mL) in the current study is much lower than the one identified in the Hong Kong study (178.2 pg/mL) [8], which may due to the fact that Hong Kong study used primarily fasting specimens, while the current study used mostly non-fasted samples. Since FGF-21 is not a routinely measured biomarker in the clinical practice yet, and the sensitivity (75.7%) and specificity (41.4%) of the best cut-off value were low in the current study, more studies are needed to validate the clinical potential of using FGF-21 in diabetes prediction in daily practice.

The strength of the current study is the adjustment for well-established diabetes risk factors (including liver enzymes), and using comprehensive statistical methods (AUC, NRI and IDI) to explore the predictive utility of FGF-21. Furthermore, we measured HbA_1c_ in the blood collected at baseline, and excluded those with HbA_1c_ > 6.5% (48 mmol/mol) to avoid undiagnosed diabetes cases. However, there are some limitations as well. First, we measured FGF-21 only once, and this one-time measurement may not represent long-term profile. However, this could lead to non-differential misclassification of FGF-21 status and thus may underestimate the true association. In addition, some information such as height, weight, and history of hypertension were self-reported, and we did not have information on family history of type 2 diabetes at baseline, thus residual confounding may exist. Furthermore, more than 70% of blood samples were non-fasting, and therefore may influence the FGF-21 levels. However, we have adjusted for fasting status in the models and no significant interaction was found with fasting status, indicating that fasting status did not influence the associations in the present study. Moreover, the sample size in the stratified analyses was small and studies with larger sample size are needed to further explore whether the association could be modified by other variables. Finally, the current study used matched case-control study design, and although it is valid in studying associations, recent studies pointed out that it may introduce bias when studying predictive utility of biomarkers [37, 38].

## Conclusions

In conclusion, we observed a strong, dose-dependent association between serum FGF-21 levels and increased risk of incident type 2 diabetes in Chinese women but not in men. Further researches are needed to validate the findings, to investigate the underlying biological mechanisms, and to examine the feasibility of targeting FGF-21 through pharmacological interventions to reduce the risk of diabetes in high-risk population.

## Additional file

Additional file 1: Table S1. The pair-wise Pearson correlation coefficients between FGF-21, age, blood levels of liver enzymes, lipids, high-sensitivity C-reactive protein and adiponectin among case and control participants, the Singapore Chinese Health Study. **Table S2.** Reclassification of type 2 diabetes cases and controls among female participants with no risk categories based on their serum concentrations of FGF-21, the Singapore Chinese Health Study. **Figure S1.** Flowchart of the Singapore Chinese Health Study. (DOCX 106 kb)

## Abbreviations

AIC: Akaike information criteria
ALT: Alanine transaminase
AUC: Area under receiver-operating characteristic curve
BMI: Body mass index
CI: Confidence interval
FGF-21: Fibroblast growth factor 21
GGT: Gamma-glutamyltransferase
HbA1c: Hemoglobin A1c
HDL-C: High-density lipoprotein cholesterol
Hs-CRP: High-sensitivity C-reactive protein
IDI: Integrated discrimination improvement
NAFLD: Nonalcoholic fatty liver disease
NRI: Net reclassification improvement
OGTT: Oral glucose tolerance test
OR: Odds ratio
ROC: Receiver-operating characteristic
SD: Standard deviation
TC: Total cholesterol
TG: Triglycerides
