# Supplementary material for: Sex-specific association between fibroblast growth factor 21 and type 2 diabetes: a nested case-control study in Singapore Chinese men and women
Source: Nutr Metab (Lond). 2017 Sep 30;14:63. doi: 10.1186/s12986-017-0216-0 (PMC5622539; doi:10.1186/s12986-017-0216-0)

**Sex-specific association between fibroblast growth factor 21 and type 2 diabetes in Singapore Chinese men and women**

Wang YL, Yuan JM, Koh WP, Pan A

**Online Supplemental Material**

The following materials are included in the Online Supplemental Material.

1. Tables S1 and S2
2. Figure S1

**Table S1.** The pair-wise Pearson correlation coefficients between FGF-21, age, blood levels of liver enzymes, lipids, high-sensitivity C-reactive protein and adiponectin among case and control participants, the Singapore Chinese Health Study

**Table S2.** Reclassification of type 2 diabetes cases and controls among female participants with no risk categories based on their serum concentrations of FGF-21, the Singapore Chinese Health Study

**Figure S1.** Flowchart of the Singapore Chinese Health Study

**Table S1.** The pair-wise Pearson correlation coefficients between FGF-21, age, blood levels of liver enzymes, lipids, high-sensitivity C-reactive protein and adiponectin among case and control participants, the Singapore Chinese Health Study

| Marker | Controls | Cases |
| --- | --- | --- |
| Age | 0.19^b^ | 0.08 |
| ALT | 0.17^b^ | 0.27^b^ |
| GGT | 0.15^a^ | 0.26^b^ |
| TC | -0.09 | -0.01 |
| HDL-C | -0.22^b^ | -0.20^a^ |
| TG | 0.16^a^ | 0.22^a^ |
| Hs-CRP | 0.13^a^ | 0.14^b^ |
| Adiponectin | -0.16^a^ | -0.25^a^ |

^a^Correlation coefficients are statistically significant at *P* <0.05;

^b^Correlation coefficients are statistically significant at *P* <0.01;

**Abbreviations:** FGF-21, fibroblast growth factor-21; ALT, alanine aminotransferase; GGT, gamma-glutamyl transferase; TC, total cholesterol; HDL-C, high-density lipoprotein cholesterol; TG, triglycerides; hs-CRP, high-sensitivity C-reactive protein.

**Table S2.** Reclassification of type 2 diabetes cases and controls among female participants with no risk categories based on their serum concentrations of FGF-21, the Singapore Chinese Health Study

| Base model 1^a^ + FGF-21 | All | Assigned to higher diabetes risk | Assigned to lower diabetes risk | NRI | | |
| --- | --- | --- | --- | --- | --- | --- |
| Expected number of event participants | 134 | 99 | 35 |  | Among event participants | 47.8% |
| Expected number of non-event participants | 134 | 75 | 59 |  | Among non-event participants | -11.9% |
|  | | | | | Overall original (95% CI) | 35.8% (11.8%, 59.8%) |
| Base model 2^b^ + FGF-21 | All | Assigned to higher diabetes risk | Assigned to lower diabetes risk | NRI | | |
| Expected number of event participants | 134 | 99 | 35 |  | Among event participants | 47.8% |
| Expected number of non-event participants | 134 | 75 | 59 |  | Among non-event participants | -11.9% |
|  | | | | | Overall original (95% CI) | 35.8% (11.8%, 59.8%) |
| Base model 3^c^ + FGF-21 | All | Assigned to higher diabetes risk | Assigned to lower diabetes risk | NRI | | |
| Expected number of event participants | 134 | 99 | 35 |  | Among event participants | 47.8% |
| Expected number of non-event participants | 134 | 73 | 61 |  | Among non-event participants | -9.0% |
|  | | | | | Overall original (95% CI) | 38.8% (14.9%, 62.8%) |

^a^Base model 1 included age (continuous) and body mass index (continuous).

^b^Base model 2 included variables in base model 1 plus smoking status (never, ever smoker), history of hypertension (yes, no), and levels of triglycerides (mmol/L), high-density lipoprotein cholesterol (mmol/L), and random glucose (mmol/L) (all in quartiles).

^c^Base model 3 included variables in base model 2 plus adiponectin (μg/mL) and high-sensitivity C-reactive protein (mg/L) (both in quartiles).

**Abbreviations:** FGF-21, fibroblast growth factor-21; NRI: net reclassification improvement.

**Figure S1.** Flowchart of the Singapore Chinese Health Study


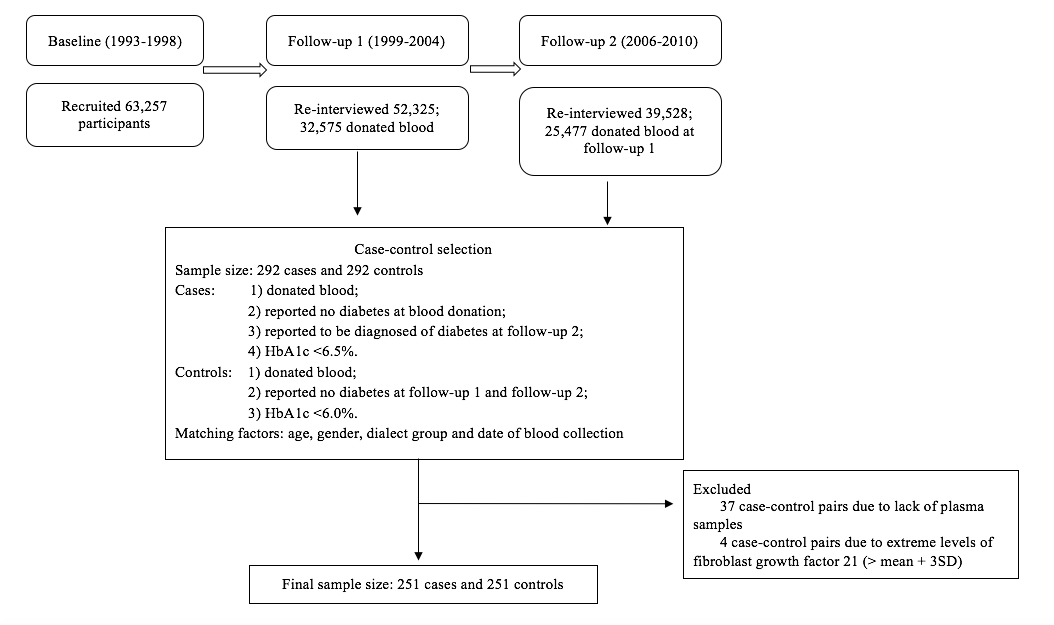

Supplement: Additional file 1: Table S1. — The pair-wise Pearson correlation coefficients between FGF-21, age, blood levels of liver enzymes, lipids, high-sensitivity C-reactive protein and adiponectin among case and control participants, the Singapore Chinese Health Study. Table S2. Reclassification of type 2 diabetes cases and controls among female participants with no risk categories based on their serum concentrations of FGF-21, the Singapore Chinese Health Study. Figure S1. Flowchart of the Singapore Chinese Health Study. (DOCX 106 kb) [file 12986_2017_216_MOESM1_ESM.docx]
